# Bioinformatics Analysis of the Factors Controlling Type I IFN Gene Expression in Autoimmune Disease and Virus-Induced Immunity

**DOI:** 10.3389/fimmu.2013.00291

**Published:** 2013-09-19

**Authors:** Di Feng, Betsy J. Barnes

**Affiliations:** ^1^Department of Biochemistry and Molecular Biology, Rutgers Biomedical and Health Sciences, Newark, NJ, USA; ^2^Rutgers Biomedical and Health Sciences, New Jersey Medical School-Cancer Center, University of Medicine and Dentistry of New Jersey, Newark, NJ, USA

**Keywords:** type I interferons, bioinformatics, autoimmunity, transcriptional regulation, transcription, genetic

## Abstract

Patients with systemic lupus erythematosus (SLE) and Sjögren’s syndrome (SS) display increased levels of type I interferon (IFN)-induced genes. Plasmacytoid dendritic cells (PDCs) are natural interferon producing cells and considered to be a primary source of IFN-α in these two diseases. Differential expression patterns of type I IFN-inducible transcripts can be found in different immune cell subsets and in patients with both active and inactive autoimmune disease. A type I IFN gene signature generally consists of three groups of IFN-induced genes – those regulated in response to virus-induced type I IFN, those regulated by the IFN-induced mitogen-activated protein kinase/extracellular-regulated kinase (MAPK/ERK) pathway, and those by the IFN-induced phosphoinositide-3 kinase (PI-3K) pathway. These three groups of type I IFN-regulated genes control important cellular processes such as apoptosis, survival, adhesion, and chemotaxis, that when dysregulated, contribute to autoimmunity. With the recent generation of large datasets in the public domain from next-generation sequencing and DNA microarray experiments, one can perform detailed analyses of cell-type specific gene signatures as well as identify distinct transcription factors (TFs) that differentially regulate these gene signatures. We have performed bioinformatics analysis of data in the public domain and experimental data from our lab to gain insight into the regulation of type I IFN gene expression. We have found that the genetic landscape of the *IFNA* and *IFNB* genes are occupied by TFs, such as insulators CTCF and cohesin, that negatively regulate transcription, as well as interferon regulatory factor (IRF)5 and IRF7, that positively and distinctly regulate *IFNA* subtypes. A detailed understanding of the factors controlling type I IFN gene transcription will significantly aid in the identification and development of new therapeutic strategies targeting the IFN pathway in autoimmune disease.

## Introduction

Patients with autoimmune diseases, such as systemic lupus erythematosus (SLE) and Sjögren’s syndrome (SS), display increased expression of type I interferon (IFN)-induced genes. Plasmacytoid dendritic cells (PDC), as natural IFN-producing cells, are considered to be a primary source of IFN-α in such diseases (1, 2). The type I IFN family consists of multiple members, including 14 IFN-α subtypes, -β, -ε, -κ, -ω, -δ, and -τ. These members may have autocrine effects on the IFN-producing cells themselves, such as PDCs, and paracrine effects on neighboring cells, as well as systemic effects on distant immune cells (3). IFNs can be added directly to cell cultures and molecular profiling performed to understand their biologic effect. For instance, the direct treatment of peripheral blood mononuclear cells (PBMCs) with 0.6 pM of IFN-α, -β, or IFN-ω led to the increased expression of about 200 genes (4). Broadly speaking, an IFN gene signature should include all of these genes. These genes can be functionally classified into antiviral pathways, apoptosis control, cell surface receptor expression, chemokine/cytokine expression, and components of IFN signaling pathways.

Although methods of bioinformatics analysis are not yet intensively used in immunology research, the field is changing fast and significant information can now be obtained from the public domain for the analysis of mechanisms controlling type I *IFN* gene expression. This report explores several elements of translational bioinformatics analysis, specifically addressing the biological questions relevant to how type I IFN expression is regulated in autoimmune disease. We collected publically available microarray gene expression datasets in Gene Expression Omnibus (GEO) at the National Center for Biotechnology Information (NCBI) and performed data mining and pathway analysis. With the growing datasets in public repository that are shared in the research community, the integrative analysis of experimental data and disease profiling data sets has become an important approach to our understanding of autoimmune disease pathology at the molecular level. In this study, we have also used human datasets from the Encyclopedia of DNA Elements (ENCODE) to understand the epigenetic codes that control the type I IFN gene cluster. This information can be used as a reference to guide future experiments that focus on epigenetic changes in more relevant human immune cell populations such as monocytes and dendritic cells. Understanding the regulation and epigenetic control of type I IFN expression will be useful for the development of new therapeutic interventions targeting the IFN pathway in autoimmune disease.

## Materials and Methods

### Materials

Gene expression microarray data were retrieved from NCBI’s GEO through series accession numbers GSE17762 and GSE10325. Data were loaded with GEO query and limma R packages from the Bioconductor project. Alternatively, GEO2R, an interactive web tool, was used. Next-generation sequencing datasets from multiple cell lines and cell types were retrieved from the ENCODE Project^1^.

### Methods

In brief, for the analysis of microarray data, gene symbols and value of log fold changes for individual genes were extracted from NCBI’s GEO and Ingenuity IPA software was used to perform pathway analysis. For next-generation sequencing datasets, ENCODE offers a few software tools for analyzing the data. One relevant tool is factor book, which organizes all the information associated with individual transcription factors (TFs) (5). Although useful, it should be noted that the current lack of information on human primary immunocytes limits one’s ability to analyze individual genes/gene clusters and therefore limits the value and/or relevance of some of these datasets.

The following information provides a brief summary of methods used for the analysis of next-generation sequencing data. For example, the epigenome analysis of the *IFNA* gene cluster was performed using a variety of resources for data visualization. In brief, the genetic region was located and retrieved in UCSC genome browser using URL http://genome.ucsc.edu/cgi-bin/hgTracks?position=chr9:21000000-21550000. Methylated/unmethylated CpGs data was retrieved using Methylation-sensitive restriction enzyme sequencing (MRE-seq) and MeDIP-seq loaded from http://genome.ucsc.edu/cgi-bin/hgTrackUi?g=ucsfBrainMethyl. Methyl Reduced Representation Bisulfite Sequencing (RRBS) tracks were loaded from http://genome.ucsc.edu/cgi-bin/hgTrackUi?g=wgEncodeHaibMethylRrbs, samples used include all cells in the following list: http://genome.ucsc.edu/cgi-bin/hgTrackUi?hgsid=342586899&c=chr9&g=wgEncodeRegTfbsClusteredV2. Histone modification data, including H3K4me3 was loaded from http://genome.ucsc.edu/cgi-bin/hgTrackUi?hgsid=342586899&c=chr9&g=wgEncodeReg. For the analysis of CTCF and other relevant TFs, we selected TFs and cell types by adding tracks from http://genome.ucsc.edu/cgi-bin/hgTrackUi?db=hg19&g=wgEncodeAwgTfbsUniform. TF binding peaks were either calculated using ENCODE pre-processed data with a False Discovery Rate of 1% or mapped to human genome hg37 using CLC Genomics Workbench software 5.5, followed by peak calling using Model-based Analysis for ChIP-Seq (MACS).

## Results and Discussion

### Relationship between the type I IFN gene signature and clinical autoimmune biomarkers

We have performed an in depth bioinformatics analysis of genes regulated by type I IFNs, as well as the mechanisms controlling type I IFN expression, in autoimmune diseases using publically available datasets. In many cases, we found that IFN-induced genes directly explain the presence of clinical biomarkers that appear in patients with autoimmune diseases. For example, we found that IFN-α increases the expression of interleukin (IL)-15 and its receptor IL-15Rα in PBMCs. IL-15, that is primarily expressed by activated monocytes and dendritic cells, binds to IL-15Rα (CD359) on accessory cells and is trans-presented to T cells that express functional IL-15Rα, composed of IL-2/15Rβ (CD122) and γc chains. Several groups have reported elevated IL-15 levels in the sera of SLE patients, however, the functional consequence of IL-15Rα activation in SLE remains to be studied (6). In addition to IL-15 and IL-15Rα, IFN-β moderately upregulates *IL-7* and *CD59* transcripts in PBMCs. IL-7 is a survival factor for naïve, early effector, and memory CD4^+^ and CD8^+^ T cells. It is primarily produced by fibroblastic reticular cells (FRCs), a mesenchymal cell population found in the stromal environment of lymphoid organs. In SLE patients, soluble (s)IL-7R concentrations were found to be elevated in the serum and raised levels of sIL-7 were detected in patients with lupus nephritis (LN) that reflected activation of kidney tissue cells (7). Receptor blockade by anti-IL-7Rα in MRL-*Fas^lpr^* lupus mice resulted in alleviation of dermatitis, lymphadenopathy, splenomegaly, and total serum IgG2a; yet, only a marginal reduction in IgG2a autoantibodies was found (8). CD59 are glycosylphosphatidylinositol-anchored proteins with complement inhibitory properties that prevent the terminal polymerization of the membrane attack complex. Increased numbers of CD55- and CD59-lymphocytes and CD59-granulocytes were found in SLE patients as compared with controls (9).

### Pathway activation by type I IFNs

Type I IFNs may play a pathological role in autoimmune disease through their ability to regulate key signaling pathways important in the innate immune response. For instance, we found that IFN-α upregulates the expression of Toll-like receptors (*TLR*)-*3* and *TLR-7*, as well as the critical cofactor myeloid differentiation primary response protein 88 (*MyD88*). IFN-α also enhances the expression of interferon regulatory factor (*IRF*)*2*, which competitively inhibits IRF1-mediated transcriptional activation of *IFNA* and *B* genes. As compared to IFN-α, the effect of IFN-β on gene expression extends to *TLR-1*, *TRAF/TANK*, *IRF4*, and *IRF1*. We also found in our analysis that the human dual specificity mitogen-activated protein kinase kinase 5 (*MAP2K5*) can be up-regulated by IFN-α/IFN-β and mitogen-activated protein kinase kinase 8 (*MAP3K8*) can be induced by IFN-β. Since p38 MAPK acts up-stream of type I IFN-induced STAT (signal transducers and activators of transcription) 1 signaling (10, 11), the up-regulation of *MAP3K8* or *MAP2K5* may provide further hints toward the biologic effects of type I IFN on cells. For example, MAP3K8 has been shown to promote the production of tumor necrosis factor (TNF)-α and IL-2 during T lymphocyte activation. It is also known that addition of IFN-α with anti-CD3 antibodies results in enhanced T helper (Th)1 responses that associate with enhanced phosphorylation of STAT1 (12).

It is well-known that IFN-α has pro-apoptotic effects in many cancer cell types including myeloma (13), renal cell carcinoma (14), and glioma (15). It is also known that monocytes stimulated with IFN-α express functional TNF-related apoptosis-inducing ligand (TRAIL), which is capable of killing myeloma cells (16). IFN-α also increases the expression of functional FasL exclusively on natural killer (NK) cells (17). The functional clustering of genes regulated by IFN-β, using DAVID tools, revealed a number of genes that control apoptosis, including *caspase 1*, *8*, and *10*, *TRAIL* (*TNFSF10*), and *FADD [Fas (TNFRSF6)-associated via death domain]*.

### The type I IFN gene signature in SLE B cells and T cells

Disease biomarkers or disease gene signatures provide important clues for our understanding of disease pathogenesis and aid in the identification and development of new therapeutic strategies for treatment. High-throughput screening technologies, such as DNA microarrays, have been used to profile disease signatures in PBMCs from SLE patients (18), and subsequently, in specific subsets such as monocytes, neutrophils, T cells, and B cells. The presence of a type I IFN gene signature in PBMC of SLE patients has been recognized for nearly 35 years now (19). However, not all IFN-inducible genes that have been identified by *in vitro* assays can be detected *in vivo* in PBMCs isolated from SLE patients. About 20 IFN-inducible genes were consistently found to be highly expressed in PBMC from SLE patients (18). In our analysis of SLE B and T cells, we found that approximately 10 IFN-inducible genes were consistently and highly expressed. The gene transcriptional signatures that appear to overlap between cell types include *Mx1*, *ISGF-3*, *PRKR*, *IFIT1*, and *IFI44* in cells that have been either exposed to type I IFNs *in vivo* or *in vitro*. This gene signature has been used as a readout for the type I IFN bioassay and is considered a measure of the “IFN-α activity score” in patients with SLE and other inflammatory or autoimmune diseases (19, 20).

Intensive pathway analyses with KEGG^2^, BioCarta^3^, and GenMAPP^4^ have shown up-regulated activation markers on SLE T cells and genes that correlate with STAT1 expression (21). Using IPA^5^ analysis of independent datasets, we also found groups of genes in the network that strongly correlate with STAT1, suggesting a persistent and strong effect downstream of type I IFNs in SLE T cells. Furthermore, IFN response factor consensus sequences (ISREs) can be found up-stream of the start sites of each of the genes in the type I IFN gene signature. Our independent analysis also indicated groups of up-regulated genes in SLE T cells that can be modulated by STAT4. Genome-wide mapping of STAT4 and IRF 5 occupancy in immune cells from SLE patients by chromatin immunoprecipitation combined with next-generation sequencing (ChIP-seq) revealed the possible cooperation of high mobility group-I/Y, specificity protein 1, and paired box 4 with IRF5 and STAT4 in transcriptional regulation (22). As noted above, IFN-regulated pathways derived from *in vitro* data do not always align with microarray datasets obtained from primary cells of SLE patients. In this regard, short-term IFN treatment has been shown to promote apoptosis signaling via TRAIL pathways. However, anti-apoptotic signatures, including elevation of caspase 8 and FADD-like apoptosis regulator (*CFLAR*), were identified in lupus T cells (21). Our bioinformatics pathway analysis identified additional genes, such as *BIRC5*, that participate in the B cell anti-apoptotic pathway in cells isolated from SLE patients. Given that apoptosis and the clearance of apoptotic material have been implicated in SLE pathogenesis, further research detailing the in depth analysis and mapping of these anti-apoptotic pathways in PBMC subsets will be of significant importance to our understanding of SLE pathogenesis.

### Genetic landscape of the type I IFN cluster

The human type I *IFN* gene cluster spans approximately 450 kb on chromosome 9p22. *IFNB* and *IFN*ε define the boundaries of the cluster, with all other type I *IFN* genes, except *IFNk*, distributed between these borders. This gene cluster also contains *KLHL9*, which is a substrate-specific adaptor of the BCR (BTB-CUL3-RBX1) E3 ubiquitin ligase complex that functions in cell division. Studies of virus-induced type I IFN production in murine fibroblasts indicates the presence of an immediate-early response gene, *IFNA4*, which is induced rapidly and without the need for ongoing protein synthesis, and *IFNA2*, *5*, *6*, and *8*, that display delayed induction, are induced more slowly, and require cellular protein synthesis. In CpG-stimulated human PDCs, *IFNA5*, *IFNA10*, *IFNA4*, *1/13*, *21*, *14*, *16*, and *6* transcription can be detected within 2 h. *IFN21* and *IFNA16* levels are dramatically up-regulated further after 8 h suggesting an efficient positive feedback loop regulating expression of these two genes. Recent analysis of data from the ENCODE Consortium suggests that this important gene cluster may be controlled by epigenetic regulation supporting new mechanistic insight and a basis for the design of experiments focused on this aspect of type I IFN gene regulation.

#### Methylation

Indeed, there has already been significant data in the literature to support the mechanism(s) of epigenetic regulation in autoimmune diseases. In particular, DNA from SLE T cells was found to be less methylated than control DNA from normal T cells by measuring the cellular deoxymethylcytosine content (23). Interestingly, non-T cells from lupus patients displayed normal DNA methylation levels (24). Decreased DNA methyltransferase (DNMT) activity in lupus T lymphocyte nuclear proteins was considered to be responsible for the observed DNA hypomethylation in lupus T cells. Patients with lupus had significantly lower levels of *DNMT1* mRNA, but not *DNMT3A* or *DNMT3B*, as compared with healthy controls (25). A preliminary analysis of microarray data from immature monocyte-derived dendritic cells (MDDCs) revealed that they express abundant amounts of *DNMT1*, which is downregulated after LPS stimulation. The methylation status of DNA from SLE PDCs and the levels of *DNMT1* expression in this important IFN-α producing cell type are not currently known.

**Figure 1:**
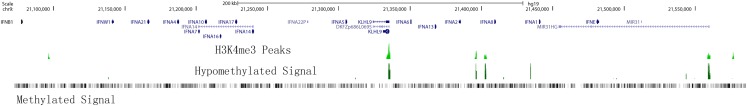
**H3K4me3 peaks and methylation tracks on the type I *IFN* gene cluster**. Members of the type I *IFN* gene cluster are shown and illustrated proportionally according to Human (*Homo sapiens*) Genome hg19. H3K4me3 peaks and UCSC DNA methylation tracks are shown for a human B cell line.

In general, hypermethylation in the promoter of a gene is associated with gene suppression, while hypomethylation is linked to gene expression; methylation within the gene body is also associated with gene expression. Two next-generation sequencing technologies have recently been developed for the analysis of gene methylation – methylated DNA immunoprecipitation sequencing (MeDIP)-seq, to detect methylated CpGs (26, 27), and MRE-seq, to detect unmethylated CpGs (28). Integrative methodologies that combine both MeDIP-seq and MRE-seq can differentiate hypermethylation, intermediate, and hypomethylation regions of DNA. An integrative analysis of *KLHL9* indicates that the CpG islands of the *KLHL9* promoter are highly hypomethylated (Figure 1). These islands are highly conserved since they were found to be present in virtually all cell types queried. Combining these data with ChIP-seq histone modification data in the same tissues, we found that hypomethylated CpGs of *KLHL9* are occupied by significant levels of trimethylated lysine 4 on histone H3 (H3K4me3) (Figure 1). Two other hypomethylated regions in the type I IFN cluster, located in the genomic region between *IFNA2* and *IFNA8*, have relative low levels of enrichment for H3K4me3 peaks (Figure 1). H3K4me3 is a histone modification that accumulates at the transcription-start site (TSS) of active genes and is believed to be important for transcription activation. Loss of H3K4me3 occurs at TSSs and leads to gene transcriptional inactivation as a result of promoter hypermethylation. The occupancy of H3K4me3 in the promoter of *KLHL9* may ensure the protection of CpG islands from methylation. In contrast, the other two hypomethylated sites that are located quite far from the TSSs of *IFNA2* and *IFNA8*, may not be functional for transcription. DNA methylation by RRBS from various cell types, including B cells, failed to reveal strong methylation signals in the *IFNA* gene cluster. One exception to this is that MeDIP-seq defined methylation peaks were found to be distributed between *IFNA* genes from brain tissue.

Thus far, data do not support that methylation is the likely major mechanism by which *IFNA* gene expression is suppressed in most non-IFN-producing cells. Further experimental studies will be necessary to determine whether constructive hypomethylation, as well as H3K4me3 occupancy, is important for regulating *IFNA* gene transcription in IFN-producing cells such as monocytes, and PDCs.

#### Chromatin structure

**Figure 2:**
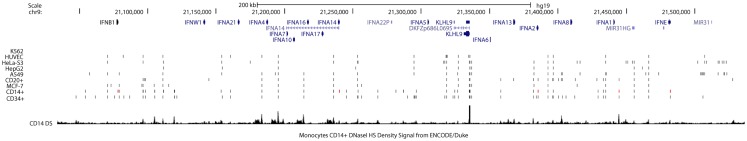
**DNase I hypersensitivity sites in the type I *IFN* gene cluster are highly conserved between cell types**. Members of the type I *IFN* gene cluster are shown and illustrated proportionally according to Human (*Homo sapiens*) genome hg19. Cell lines and cell types analyzed are listed on the left side. Short vertical lines below the gene track indicate the open chromatin position marked by DNaseI hypersensitivity sites from ENCODE/OpenChromatin (Duke University) for each cell type. Red lines indicate the novel sites identified between cell types. The DNase I hypersensitivity signal peaks for CD14^+^ monocytes are shown at the bottom for reference to chromatin marks.

There are multiple *IFNA* and *IFNB* genomic regions that have open chromatin structure in an evolutionally conserved pattern across species and most human cell types. Since DNase I hypersensitive sites (DHSs) reflect the local openness and accessibility of chromatin, chromatin structure or accessibility of *IFNA* clustering may be similar among different cells. In general, hypersensitive sites are found only in the chromatin of cells in which the associated gene is being expressed, and do not occur when the gene is inactive. Therefore, mapping DHSs within nuclear chromatin is a powerful method of identifying genetic regulatory elements (29). However, the distribution of DHSs in promoters and other gene regions of similarly expressed genes differs among different chromosomes. Furthermore, silenced genes have a more open chromatin structure than previously thought and DHSs in 3′-untranslated regions (3′-UTRs) have been shown to negatively correlate with gene expression levels (30), thus going against the standard dogma. Bioinformatics analysis of DHSs in the *IFN* gene cluster between different cell types revealed a highly conserved pattern (Figure 2); however, we found additional DHSs in CD14^+^ monocytes that can produce type I IFNs. We also found that CD34^+^ stem cells have more DHSs close to promoters within the *IFN* gene cluster (Figure 2). These data support the presence of unique cell-specific chromatin structures which may play important regulatory roles in the control of type I IFN expression.

#### Histone modification

Modifying the chromatin template at a particular gene locus can also serve as an important mechanism of gene transcriptional activation that exhibits cell-type specific expression patterns. The functional importance of histone acetylation in type I IFN production has been supported by studies that show increased IFN-β expression in cells treated with histone deacetylase inhibitors, such as Trichostatin A (TSA) (31), and decreased IFN-β expression in murine macrophages where the binding of bromodomain-containing BET (bromodomain and extraterminal) transcriptional regulators to acetylated histones was inhibited (32). Di- or tri-methylation of H3K9 is capable of suppressing gene expression not only passively, by inhibiting acetylation, but also actively, by recruiting transcriptional repressors of the heterochromatin protein 1 (HP1) family. We found that H3K9me2 occupancy at *IFN* and *ISG* promoters is inversely correlated with gene expression. Furthermore, human MDDCs that are capable of producing type I IFNs, as compared with human lung fibroblasts that do not, show decreased H3K9me2 occupancy at the *IFNB* promoter. In the absence of G9A, a methyltransferase for H3K9me2, non-professional IFN-producing cells were shown to be converted into potent IFN-β producers (33). Together, these data support the importance of histone modifications in the regulation of type I IFN expression.

**Figure 3:**
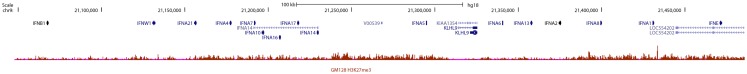
**H3K27me3 peaks on the type I *IFN* gene cluster**. Members of the type I *IFN* gene cluster are shown and illustrated proportionally according to Human (*Homo sapiens*) Genome hg19. H3K27me3 peaks were found along the entire region of the *IFN* gene cluster in the human B cell line GM128.

H3K27me3, on the other hand, are found to be associated with the repression of gene transcription in a cell-type specific manner. Polycomb Repressive Complex 2 (PRC2) is a histone methyltransferase that catalyzes tri-methylation of Histone 3 at Lysine 27 (H3K27me3) (34). A detailed profile of H3K27me3 peaks reveal that broad peaks at TSS are associated with transcriptional suppression while skewed peaks up-stream of the TSS may not be suppressive (35). Indeed, we found that *IFNA* regions in B cells, which are incapable of producing IFN-α, are widely occupied with H3K27me3, as shown by the substantive peaks found along the gene cluster (Figure 3). In contrast, ChIP-seq data from monocytes demonstrate that H3K27me3 peaks occupy some *IFNA* genes, such as *IFNA2*, *IFNA14*, and intergenic regions between *IFNA2* and *IFNA8*, while the remaining *IFNA* genes were not suppressed by H3K27me3.

As mentioned above, current dogma holds that H3K4me3 represents a chromatin landmark that is present at the TSS for genes that are either actively transcribed or permissive for gene transcription. However, H3K4me3 are not sufficient to license cells to produce IFN-α. For example, multiple H3K4me3 occupancy peaks can be identified in the IFN regulatory regions in B cells that do not express IFN-α. A good comparison would be with PDCs, yet the histone codes are not yet available for this cell type. In PDCs, TLR-7 signaling quickly turns on transcription of *IFNB*, *IFNA2*, *IFNA8*, and *IFNA14* genes at 30 min post-stimulation with peak levels being achieved at this time point. In comparison, peak levels of *IFNA5*, *IFNA6*, *IFNA10*, *IFNA13*, and *IFNA21* were observed around 4 h post-stimulation (36). Based on our bioinformatics analysis, we reason that transcriptional suppression by H3K27me3, if it exists in PDCs in a pattern similar to that found in CD14^+^ monocytes, may not be functional in PDCs or can quickly be replaced by H3K4me3 after TLR-7 activation. Alternatively, the *IFN* gene cluster in PDCs may not have H3K27me3 markers. It is not known whether chromatin change is necessary for *IFNA* transcriptional activation or whether chromatin status is responsible for differentially transcribed type I *IFN* genes. Further studies in human PDCs will be required to address this.

#### Transcription factors regulating basal repression of IFNA gene expression

**Figure 4:**
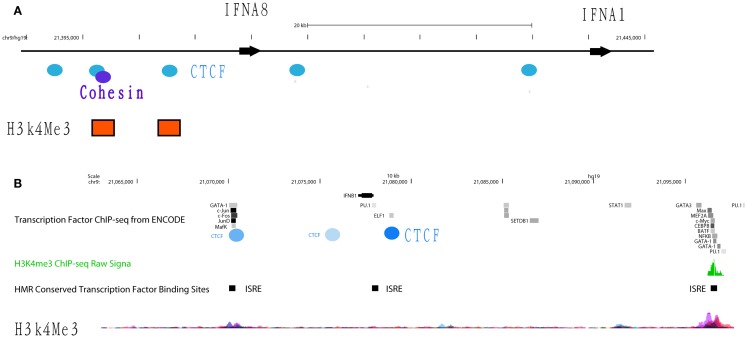
**Differential occupancy for insulators CTCF and cohesin on the *IFNA8* and *IFNB* genes**. **(A)** The genomic regions of *IFNA8* and *IFNA1* are shown proportionally according to Human (*Homo sapiens*) Genome hg19. CTCF (blue circles) and cohesin (purple circles) occupancy positions are marked according to transcription factor ChIP-seq datasets from ENCODE. Orange squares indicate positions enriched with H3K4Me3 signals. **(B)** The genomic region of *IFNB* is shown with transcription factor binding sites from the ENCODE database. Experimentally verified CTCF binding sites are shown by the blue circles, with the darker shades of blue denoting signal intensity; light blue – low intensity, dark blue – high intensity. IFN-stimulated response elements (ISRE) are labeled with black squares. Peaks showing H3K4me3 signals are shown.

The transcriptional repressor CTCF (11-zinc finger protein) or CCCTC-binding factor is thought to regulate the 3-dimensional (3D) structure of chromatin by binding strands of DNA together and forming DNA loops (37). CTCF represses gene expression by blocking the interaction between enhancers and promoters (38). This phenomenon may serve as a chromatin barrier to block the spread of heterochromatin structures and set boundaries between active chromatin regions marked by histone H2A acetyl Lys5 (H2AK5ac) and repressive regions marked by H3K27me3 (39). The cohesin complex, consisting of cohesion proteins SMC1, SMC3, SCC3, and the α-kleisin SCC1, may contribute to CTCF-mediated repression. Many CTCF/cohesin binding sites are located at promoter regions suggesting a joint regulatory role for these factors (40). Although most cohesin sites overlap with CTCF, a significant proportion of each factor’s sites are independent of the other, implying CTCF-independent functions of cohesin as well as cohesin-independent CTCF functions. Bioinformatics analysis of CTCF ChIP-seq data from ENCODE cell lines identified several CTCF insulators that are basally located in the promoters and intergenic regions of *IFNA5*, *A1*, *A2*, and *A8* (Figure 4A). In gum fibroblast cells (AG09319), we found five CTCF binding sites in the region covering *IFNA14*, *A17*, *A16*, *A10*, *A7*, and *A4*. There is only one CTCF/SMC3 binding site in the *IFNB* gene. The regulatory region of the *IFNA2* gene contains two CTCF binding sites. The second CTCF site yields co-binding with SMC3, suggesting the cohesin complex may function in this *IFN* genomic region. Based on these data, we speculate that CTCF may indeed function as an *IFNA* suppressor and block promoter activation.

Within the *IFNA* gene cluster, we have yet to identify any other TF in the ENCODE datasets that basally occupies the promoter regions between TSSs and the proximal CTCF sites. In contrast, multiple TFs, such as NF-κB and PU.1 (in B cells), do constitutively occupy regions up-stream of CTCF sites that control individual *IFNA* genes. CTCF binding sites are not conserved but cell-type specific. While the majority of cells show CTCF occupancy up-stream of the *IFNA2* gene, binding is absent in fibroblast cells. Similarly, at the *IFNB* promoter, CTCF binding was identified in some B cells lines, HeLa cells, MCF-7, and osteoblast cells, but not in any fibroblast cell lines or A549 lung carcinoma cells. Lack of binding of this insulator may render fibroblast cells to produce type I IFNs upon the appropriate stimulation, such as viral infection, thus supporting that CTCF binding to the *IFNA* gene may be regulated. In this regard, dexamethasone treatment in A549 cell lines induces CTCF to bind to the *IFNA8* promoter. Finally, the discrepancy of CTCF binding patterns in Epstein–Barr virus (EBV)-transformed B cell lines suggests that viral infection may interfere with CTCF function. It is known that CTCF/cohesin occupancy is essential for IFN-gamma (*IFNg*) gene transcription (41). Thus, this complex may have a similar function and be important for regulating *IFNB* gene transcription via maintaining the 3D chromatin structure of the *IFNB* locus in fibroblast cells (Figure 4B). Based on these data, we propose that the DNA regions in the *IFN* gene cluster that contain CTCF occupancy may be subject to control by this factor to ensue *IFNA* transcription during viral or viral-like challenges in IFN-producing cells. This region may also be used as a landmark to demarcate the promoter region that spans from a TSS to the CTCF binding sites and enhancer regions located up-stream of CTCF binding site.

#### Transcription factors that regulate induction of IFNA gene expression

**Figure 5:**
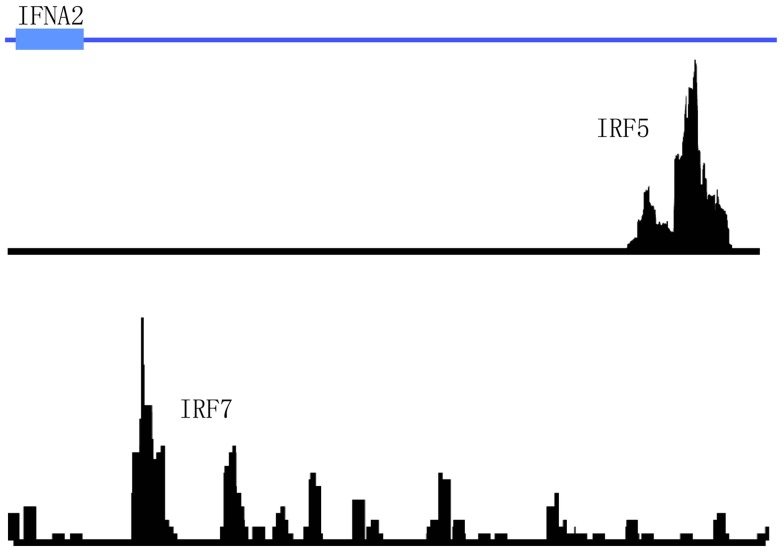
**Differential binding of IRF5 and IRF7 to the *IFNA2* gene in human primary PDCs stimulated with virus**. The genomic region of *IFNA2* is shown with IRF5 and IRF7 ChIP-seq peaks plotted according to their enrichment positions. Briefly, human primary PDCs were stimulated with Herpes simplex virus (HSV) for 4 h and cells cross-linked and harvested for immunoprecipitations with anti-IRF5 or anti-IRF7 antibodies.

Interferon regulatory factors, as their name suggests, have been long known to regulate type I *IFN* gene expression (42). Of the nine mammalian IRF family members currently identified to date, IRF7 has garnered the most attention for its role in regulating *IFNA* gene expression (3). IRF7 is highly expressed in human PDCs and allows bypass of the classic autocrine feedback loop that is regulated by IFN-β (43). IRF7 was also shown to be required for murine PDCs to produce an antiviral IFN immune response (44). Similarly, IRF5 has been implicated in the regulation of type I *IFN* gene expression (45). Early data in human cell lines revealed the regulation of type I *IFN* genes and IFN-stimulated genes (ISGs) by IRF5 in response to virus (46). Later data in mice supported these findings. For example, splenic PDCs from mice lacking *Irf5* were shown to produce less type I IFNs in response to virus infection (47). IRF5 has also been recently reported to regulate IFN-β production in myeloid dendritic cells downstream of the mitochondrial antiviral-signaling protein (MAVS) (48). Furthermore, recent studies demonstrate that IRF5 and NF-κB p50 are key co-regulators of IFN-β and IL-6 expression in TLR9-mediated activation of human PDCs (49). Although both of these IRF family members have been implicated as key regulators of IFN-α production, no ChIP-seq data is available to support these findings. Interestingly, data from the aforementioned STAT4/IRF5 ChIP-seq datasets in PBMCs did not support the direct regulation of type I IFN expression by IRF5 since no peaks were detected in the IFN gene cluster after immunoprecipitation with anti-IRF5 antibodies (22). In this case, PBMCs were stimulated with either IFNa2 or SLE immune complexes before immunoprecipitation with anti-IRF5 or anti-STAT4 antibodies. In the case of IRF7, a cursory review of the literature and publicly available datasets indicate that no ChIP-seq data is currently available for this TF. We have recently performed IRF5 and IRF7 ChIP-seq in human PDCs stimulated with virus. Our unpublished data indicate that these two TFs bind to different regions in the *IFNA* gene cluster (Figure 5). These data support the distinct and differential roles for IRF5 and IRF7 in type I *IFN* gene regulation (45, 49). With regard to autoimmune diseases such as SLE and SS that display a pathogenic type I *IFN* gene signature, determination of the mechanisms by which these two IRF family members cooperatively and distinctly regulate *IFNA* subtype expression in the critical IFN-α producing cell types will be important for the design of new therapeutic strategies targeting these two factors.

## Concluding Remarks

With the recent generation of large datasets in the public domain from next-generation sequencing and DNA microarray experiments, others and we have begun to perform detailed analyses of cell-type specific gene signatures as well as identify distinct TFs that differentially regulate these gene signatures in a cell type- and disease-specific manner. This report describes a sample workflow and method of integrative analysis to inspect, clean, and model data from GEO and ENCODE with the goal of highlighting information and knowledge discovery at the gene cluster level. We demonstrate that this method can extract valuable information including downstream pathway analysis, DNA methylation, chromatin structure, histone modification, and TF binding to a gene of interest (in our case, type I *IFNA*s). This report summarizes data from our bioinformatics analysis of the type I *IFN* gene cluster using data in the public domain and experimental unpublished data from our lab (Tables 1 and 2). We have found that the genetic landscape of the *IFNA* and *IFNB* genes are occupied by TF, such as insulator CTCF and cohesin, that negatively regulate transcription, as well as IRF5 and IRF7, that positively and distinctly regulate the *IFNA* subtypes. This information can be used as a reference to guide future experiments that focus on proving and/or disapproving these novel regulatory mechanisms that control type I *IFN* expression. A detailed understanding of the factors controlling type I *IFN* gene transcription will significantly aid in the identification and development of new therapeutic strategies targeting the IFN pathway in autoimmune disease.

**Table 1:** **Results from computational pathway analysis of microarray data sets**.

Genes and pathways	*Ex vivo* type I IFN treatment	In SLE patients
IL-15 and its receptor IL-15Rα	Up-regulated	Up-regulated
IL-7	Up-regulated	Up-regulated
CD59	Up-regulated	Up-regulated
MAP kinase	MAP kinase (ERK2) activity at up-stream of STAT1, MAP2K5, MAP2K5 are up-regulated	Unknown
TLR pathway (*TLR-3, 7, 1*, *TRAF/TANK*, *IRF4*, and *IRF1)*	Up-regulated	TLR-7 up-regulated^a^
STAT	STAT1	STAT1, STAT4
Apoptotic pathways	Up-regulated *caspase 1*, *8*, and *10*, *TRAIL, FADD*	Up-regulated anti-apoptotic genes including *BIRC5*

^a^ Indicates data from Ref. (50).

The following list of genes and pathways were predicted to be active in PBMCs treated with type I IFN *ex vivo* and in SLE patients.

**Table 2:** **Results from the computational analysis of ENCODE next-generation sequencing data on the type I *IFN* gene cluster**.

Epigenetic markers	Factors that affect type I IFN gene cluster
Chromatin structure	Monocytes display more DNase I hypersensitivity sites within gene cluster
Methylation	Methylation not found in non-IFN-producing cells; hypomethylated CpG island identified in cluster
Histone modification	H3K4Me3, H3K27me3, H3K9me2
Conserved transcription factor binding site	HMR conserved transcription factor binding sites computed with the Transfac Matrix Database (v7.0) identified ISRE sites
Transcription factors	IRF5, IRF7
Insulator	CTCF, SMC3, cohesin complex

## Conflict of Interest Statement

The authors declare that the research was conducted in the absence of any commercial or financial relationships that could be construed as a potential conflict of interest.

## Footnotes

^1^http://genome.ucsc.edu/ENCODE/

^2^www.genome.ad.jp

^3^www.biocarta.com

^4^www.genmapp.org

^5^www.ingenuity.com
